# Blue Light-Induced Intracellular Movement of Phototropins: Functional Relevance or Red Herring?

**DOI:** 10.3389/fpls.2016.00827

**Published:** 2016-06-09

**Authors:** Emmanuel Liscum

**Affiliations:** ^1^Division of Biological Sciences, University of MissouriColumbia, MO, USA; ^2^Christopher S. Bond Life Sciences Center, University of MissouriColumbia, MO, USA

**Keywords:** phototropin, blue-light, photoreceptors, phototropism, chloroplast movement, Golgi, protein trafficking, endocytosis

The phototropin blue light (BL) photoreceptors, phot1 and phot2, mediate a number of organellar, cellular, and tissue/organ-level responses in plants including: phototropism, chloroplast accumulation and avoidance, stomatal opening, leaf expansion, and petiole/leaf orientation (Liscum et al., 2014). Not surprisingly, given the contribution of these responses to the optimization of photosynthetic efficiency and plant growth, several studies have described an intimate relationship between phot function and plant fitness (Galen et al., 2004, 2007a,b; Takemiya et al., 2005; Goh et al., 2009; Crepy and Casal, 2015). So how do phots induce such a wide variety of critical physiological responses? Though this remains a largely unanswered question, one apparent common early response has received recurrent attention; namely, BL-induced intracellular movement of phots (Sakamoto and Briggs, 2002; Knieb et al., 2004; Kong et al., 2006, 2007, 2013a,b; Aihara et al., 2008; Han et al., 2008; Kong and Nagatani, 2008; Sullivan et al., 2008, 2010, 2016b; Wan et al., 2008; Zienkiewicz et al., 2008; Kaiserli et al., 2009; Ding et al., 2011; Aggarwal et al., 2014; Kami et al., 2014; Preuten et al., 2015; Ishishita et al., 2016). Key observations made during these studies will be discussed here, keeping always in mind an important question: Does, or will, the study of phot movement improve our fundamental understanding of phot function, or are these events merely a red herring?

Phots are hydrophilic and contain no obvious membrane-spanning domain (Huala et al., 1997; Jarillo et al., 2001; Kagawa et al., 2001), yet it has long been known from biochemical fractionation studies that both phot1 and phot2 associate with the inner face of the plasma membrane in etiolated and dark-adapted plants (Gallagher et al., 1988; Palmer et al., 1993; Salomon et al., 1996; Harada et al., 2003; Knieb et al., 2004). Sakamoto and Briggs utilized Arabidopsis plants expressing a functional phot1 tagged with GFP to demonstrate that a fraction of phot1 is “released” from the plasma membrane to the cytoplasm in response to BL exposure (Sakamoto and Briggs, 2002). A subsequent study focused exclusively on etiolated seedlings and found that, with the exception of stomatal cells, all cells expressing the phot1-GFP protein exhibited BL-induced movement of the receptor from the plasma membrane to a “distinct mosaic pattern” within the cytoplasm (Wan et al., 2008). Moreover, the movement of phot1-GFP exhibits properties consistent with a first-order photoreaction and occurs within minutes at high fluences of BL (Wan et al., 2008). Examples of BL-induced movement of phot1-GFP in etiolated Arabidopsis seedlings are shown in Figure 1. Cell fractionation studies have confirmed the BL-induced movement of both native and GFP-tagged phot1 from the plasma membrane to cytosolic locations (Sakamoto and Briggs, 2002; Knieb et al., 2004; Sullivan et al., 2008). Though the Sakamoto and Briggs study clearly represents the beginning of our collective fascination with the intracellular movement of phots, it is worth noting that the question of “function or red herring?” was first considered by these authors: “…it remains to be determined if this light-activated shift in subcellular localization plays a signaling role, and if so, with respect to which responses” (Sakamoto and Briggs, 2002).

**Figure 1:**
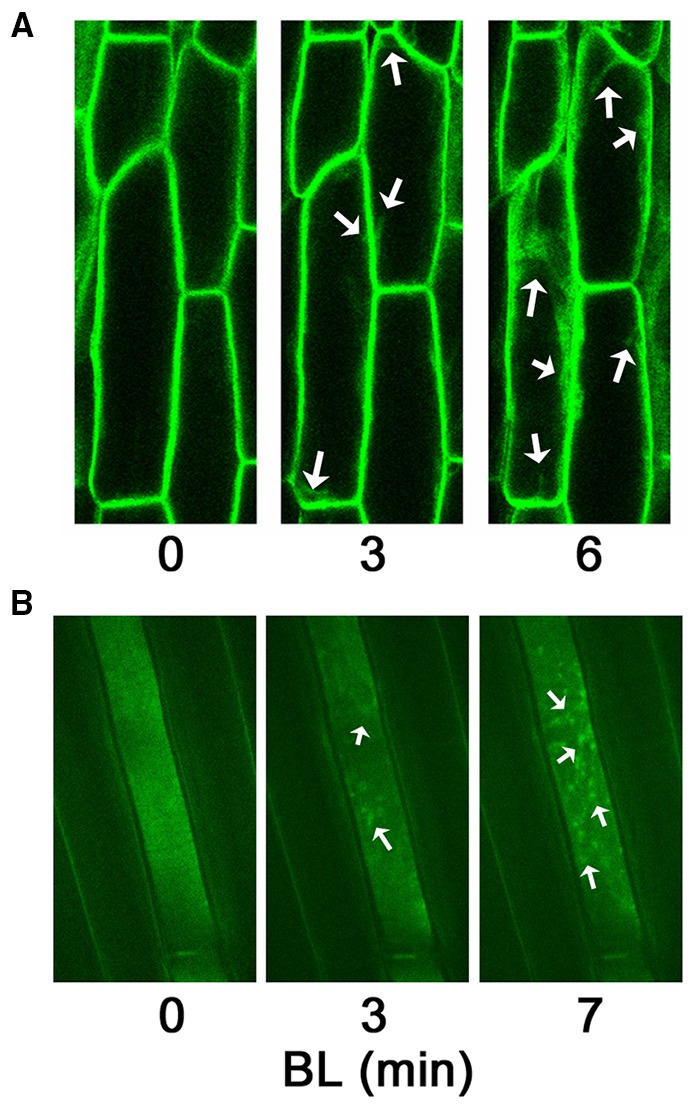
**Blue light-induced movement of phot1-GFP in etiolated hypocotyl cells of Arabidopsis**. These images are presented as representative images of what has been observed by numerous groups many times previously. **(A)** Inverted confocal microscopy (Zeiss LSM 510 META) of cortical cells within the elongation zone just below the apical hook. Images are brightest point projections from midway through the cell. After identifying cells to be imaged under low intensity blue laser light (488 nm), an image was taken (time 0), and then the cells were exposed to moderate intensity blue laser light (7 μmol m^−2^ s^−1^) for the indicated times. Note, the uniform plasma membrane localization at time 0. Arrows indicate some of the most prominent areas of cytosolic phot1-GFP moving from the plasma membrane. **(B)** Spinning disk confocal microscopy (Olympus IX-71 with Yokogawa spinning disk) of cortical cells within the elongation zone just below the apical hook. The focal point of the cell in the center of the image is the inner face of the plasma membrane. After identifying the cell and position to be imaged under green laser light (515 nm), an image was taken (time 0), and then the cells were exposed to moderate intensity blue laser light (488 nm) for the indicated times. Note the rather uniform distribution of phot1-GFP along the surface of the plasma membrane at time 0, but punctate aggregation and formation of mosaics (arrows) after BL exposure.

Not surprisingly similar experiments with plants expressing phot2-GFP soon followed. As was observed for phot1 (Sakamoto and Briggs, 2002), a vast majority of phot2-GFP protein is found associated with the plasma membrane, but a fraction of the total pool is relocalized to the cytosol in response to BL (Kong et al., 2006). Although some cytoplasmic phot2-GFP is also found in a “distinct mosaic pattern,” like phot1 (Sakamoto and Briggs, 2002; Kong and Nagatani, 2008; Kaiserli et al., 2009), a majority is found associated with the Golgi apparatus (Kong et al., 2006, 2007, 2013a). Aggarwal and colleagues have suggested that Golgi-associated phot2 may not be translocated from the plasma membrane *per se*, but rather from a small cytoplasmic fraction of phot2 that already exists prior to BL irradiation (Aggarwal et al., 2014). Kong and colleagues have also found that BL exposure results in some phot2 (and to a lesser extent, phot1) associating with the chloroplast outer membrane (Kong et al., 2013b).

Whereas the aforementioned studies with GFP-tagged phot1 and phot2 provided no connection between BL-induced internalization of phots and physiological function, they were sufficiently intriguing to prompt a number of additional studies aimed at defining the biochemical and cell biological parameters governing phot localization. First, it appears that the carboxyl-terminal portion of the protein kinase domain (PKD) of a phot is necessary for proper association with the plasma membrane, Golgi membrane, and chloroplast outer membrane (Kong et al., 2007, 2013a,b). For example, a phot1 protein lacking the carboxyl-terminal most 35 amino acids (designated phot1ΔC35) exhibits largely diffuse cytoplasmic localization independent of light condition (Kong et al., 2013a). A similarly truncated phot2 protein (phot2ΔC42) not only exhibits constitutive cytoplasmic localization, but also fails to relocalize to either the Golgi or chloroplast outer membrane in response to BL (Kong et al., 2013a,b). Second, though the PKD is necessary for membrane-association, kinase activity is not (Kong et al., 2007; Kaiserli et al., 2009). On the other hand, PKD activity does appear essential for BL-induced relocalization of phot1 from the plasma membrane to the cytoplasm (Kaiserli et al., 2009), and for phot2 association with the Golgi (Kong et al., 2006, 2007). In the case of phot1 at least, it is not merely PKD activity but receptor autophosphorylation that is necessary for relocalization (Kaiserli et al., 2009). Results with a photoactive, but kinase-dead, full-length and PKD-only phot2 proteins lead Aggarwal and colleagues to propose that it is not kinase activity, but rather the conformational changes associated with light activation of the receptor that are required for BL-induced Golgi-association of phot2 (Aggarwal et al., 2014). It is however worth noting that the latter studies were done in a *Nicotiana benthamiana* transient expression system (Aggarwal et al., 2014) where the kinase-dead Arabidopsis phot2 being assayed might have been phosphorylated in *trans* by a native tobacco phot in response to BL exposure, as can occur with phot1 (Kaiserli et al., 2009), thus confounding interpretation of the observed results. Third, it was found that both phot1 and phot2 associate with clathrin at the plasma membrane (Kaiserli et al., 2009; Roberts et al., 2011; Aggarwal et al., 2014), raising the possibility that BL-induced movement of phots from the plasma membrane occurs via clathrin-mediated endocytosis. Results from pharmacological studies are consistent with this hypothesis for phot1 (Kaiserli et al., 2009), but not phot2 (Aggarwal et al., 2014). Though intuitively the latter results seem at odds with the findings that phot2 associates with the Golgi in response to BL (Kong et al., 2006, 2007, 2013a), they are not inconsistent with the proposal that it is cytoplasmic, rather than plasma membrane-associated, phot2 that translocates to the Golgi (Aggarwal et al., 2014). Studies of phot turnover suggest that clathrin-associated phot1 and phot2 may represent sub-pools of the receptor targeted for degradation, either as internalized protein or at the plasma membrane itself (Kaiserli et al., 2009; Sullivan et al., 2010; Roberts et al., 2011; Aggarwal et al., 2014). As much as these studies help refine our understanding of the BL-induced intracellular movement of phots, we are left with the same nagging question: Are these events functionally relevant or are we just chasing a red herring?

The Briggs laboratory was the first group to tackle the hard functional question. It has been known for some time that red light (RL) pre-treatment of etiolated Arabidopsis seedlings induces a phytochrome A (phyA)-dependent enhancement of subsequent BL-induced phot1-mediated phototropism (Parks et al., 1996; Janoudi et al., 1997; Stowe-Evans et al., 2001; Whippo and Hangarter, 2004; Sullivan et al., 2016a). Han and colleagues found that in seedlings pre-irradiated with RL, phot1 is no longer mobilized from the plasma membrane in response to BL irradiation (Han et al., 2008). Moreover, this retention of phot1 at plasma membrane requires phyA and exhibits the photobiological hallmarks associated with phyA-dependent enhancement of phototropism (Han et al., 2008). Based on these findings the authors proposed that increased amounts of photoactive phot1 at the plasma membrane could positively impact lateral auxin transport to facilitate enhanced phototropism (Han et al., 2008). While not expressly stated, the results from this study also imply that cytoplasmic phot1 is not likely functional, at least with respect to phototropism.

Rather than attempting to correlate phot1 movement with a particular physiological response, Preuten and colleagues recently took a different approach by asking: What happens to phot responses if phot1 is irreversibly tethered to the plasma membrane (Preuten et al., 2015)? Phot1 modified with a myristoylation- or farnesylation-tag associates with the plasma membrane and does not exhibit BL-induced relocalization to the cytoplasm (Preuten et al., 2015). Expression of either of these recombinant phot1 proteins in a *phot1phot2* double mutant results in complementation of phot1-dependent responses, including phototropism, petiole positioning, leaf flattening, and chloroplast accumulation (Preuten et al., 2015). Interestingly, RL-induced enhancement of phototropism was found to still occur in these phot1-tethered lines (Preuten et al., 2015), suggesting that the conclusions drawn by Han and colleagues about phyA-dependent retention of phot1 at the plasma membrane being potentially causative for phototropic enhancement (Han et al., 2008) may be incomplete. However, both studies implicate plasma membrane-associated phot1 as the functionally active pool. Preuten and colleagues also found that BL-induced turnover of phot1 is normal in these transgenic lines (Preuten et al., 2015), suggesting that if clathrin-associated phot is targeted for degradation (Kaiserli et al., 2009; Sullivan et al., 2010; Aggarwal et al., 2014), that degradation occurs at the plasma membrane. At present there is no reason to expect that phot1 would need to be internalized to be degraded as even the CRL3^NPH3^ ubiquitin ligase complex that marks phot1 for BL-dependent turnover resides at the plasma membrane (Roberts et al., 2011).

It would appear, based on results from Preuten et al. (2015), that the overarching question we wished to address here can be answered with a resounding: “RED HERRING.” However, at least one well-characterized phot2 response defies this conclusion. Kong and colleagues have shown that the chloroplast avoidance response to high intensity BL (Jarillo et al., 2001; Kagawa et al., 2001; Sakai et al., 2001; Kong and Wada, 2016) requires association of phot2 with the plasma membrane, Golgi membrane and chloroplast outer membrane (Kong et al., 2013b). It is association with the chloroplast outer membrane in particular, which occurs only in response to BL, that connects phot2 movement with a functional outcome (Kong et al., 2013b). As if to provide a new “siren song” to an already intrigued community, Kong and colleagues concluded: “The different intracellular localization of phototropins and/or their interactors with cellular components would be important for the precise regulation of [these] responses under fluctuating environmental light conditions” (Kong et al., 2013b).

We close by again asking: Does, or will, the study of phot movement improve our fundamental understanding of phot function, or are these events merely a red herring? With respect to phot1, the current evidence suggests we have wandered far down an unfulfilling path toward the “red herring,” while a functional role for phot2 relocalization appears alive. Still, the commonality of the BL-induced movement responses of phots across cell, tissue, and organ types, as well as complexity of the mechanisms regulating the responses, piques the reductionist's curiosity. Investigators will need to decide if the seduction of finding meaning in phot movement is worth the risk of continuing such studies, or if persistence is tantamount to joining the mythical hounds trained on smoked kippers who gleefully follow the scent down the rabbit hole that only leads to a potential nowhere. What about this investigator? Well, one can never meet a Cheshire Cat or Mad Hatter (Carroll, 1865) if one never risks the unknown.

## Author contributions

The author confirms being the sole contributor of this work and approved it for publication.

## Funding

Research in the Liscum laboratory is supported by the National Science Foundation (IOS-1146142).

### Conflict of interest statement

The author declares that the research was conducted in the absence of any commercial or financial relationships that could be construed as a potential conflict of interest.
